# EIF2α–ATF4–CHAC1 Signalling Links ER Stress to Ferroptosis in Human Aortic Smooth Muscle Cells: Mechanistic Insights and Therapeutic Implications

**DOI:** 10.1111/jcmm.71104

**Published:** 2026-05-02

**Authors:** Changwen Fang, Xiaolong Du, Yadan Wang, Yiqi Jin, Wenbin Wang

**Affiliations:** ^1^ Department of Vascular Surgery The Second Affiliated Hospital of Anhui Medical University Hefei China; ^2^ Department of Vascular Surgery and Intervention The Affliated Suzhou Hospital of Nanjing Medical University Suzhou China; ^3^ Department of Vascular Surgery, Affiliated Drum Tower Hospital Medical School of Nanjing University Nanjing China; ^4^ Department of Operating Room The Affliated Suzhou Hospital of Nanjing Medical University Suzhou China

**Keywords:** EIF2α–ATF4–CHAC1, endoplasmic reticulum stress, ferroptosis, GPX4, vascular smooth muscle cells

## Abstract

Ferroptosis—a regulated, iron‐dependent cell death driven by lipid peroxidation—has been implicated in vascular pathology, yet how endoplasmic reticulum (ER) stress couples to ferroptotic execution in vascular smooth muscle cells (VSMCs) remains unclear. We investigated whether the PERK–EIF2α–ATF4–CHAC1 signalling axis links ER stress to ferroptosis in human aortic SMCs. Human aortic smooth muscle cells (HASMCs) and primary aortic smooth muscle cells (AoSMCs) were exposed to erastin (± ferrostatin‐1), with pharmacologic modulation using buthionine sulfoximine (BSO; glutamate–cysteine ligase inhibitor) and salubrinal (selective EIF2α dephosphorylation inhibitor). Viability (CCK‐8), ER‐stress markers (p‐PERK, p‐EIF2α, GRP78, ATF4, CHAC1) and ferroptosis effectors (GPX4, ACSL4, ALOX15) were quantified by Western blotting and densitometry. Immunofluorescence assessed GPX4 and p‐EIF2α/CHAC1 colocalization. Lipid peroxidation was evaluated by C11‐BODIPY 581/591 imaging and malondialdehyde (MDA) content; labile Fe^2+^ was measured by calcein‐AM quenching; apoptosis was examined by TUNEL. Statistics used *t*‐tests or one‐way ANOVA. Erastin reduced viability and activated the EIF2α–ATF4–CHAC1 pathway in both HASMCs and AoSMCs, as evidenced by increased p‐EIF2α/ATF4/CHAC1 and decreased GPX4, with concomitant upregulation of ACSL4 and ALOX15. GPX4 immunofluorescence declined, while C11‐BODIPY and MDA indicated robust lipid peroxidation, and calcein‐AM revealed expansion of the labile Fe^2+^ pool; ferrostatin‐1 mitigated these changes. BSO further amplified p‐PERK→p‐EIF2α → ATF4 → CHAC1 signalling and deepened GPX4 loss, whereas salubrinal sustained EIF2α phosphorylation, augmented ATF4/CHAC1, and further depressed GPX4. Across conditions, effects were consistent in both cell types and reached statistical significance (most *p* < 0.05 to < 0.01). ER‐stress signalling via PERK–EIF2α–ATF4–CHAC1 constitutes a proximal driver of ferroptosis in human aortic SMCs by eroding glutathione tone and disabling GPX4, thereby promoting lipid peroxidation in an iron‐rich milieu. Pharmacologic tuning of EIF2α phosphorylation and glutathione biosynthesis modulates ferroptotic susceptibility, nominating EIF2α–ATF4–CHAC1, GPX4/GSH homeostasis and iron handling as actionable nodes to preserve VSMC integrity in oxidizing vascular environments.

## 1 Introduction

Ferroptosis is an iron‐dependent, non‐apoptotic mode of regulated cell death driven by unchecked lipid peroxidation of polyunsaturated phospholipids [1]. Unlike apoptosis or necroptosis, ferroptosis is morphologically and biochemically defined by glutathione peroxidase 4 (GPX4) inactivation, reactive iron accumulation and the propagation of phospholipid hydroperoxides that ultimately compromise membrane integrity [2]. In their earlier research, Wang et al. discovered that a gastric cancer prognosis model constructed based on ferroptosis‐related genes possesses stable and reliable predictive efficacy, effectively evaluating the prognostic outcomes and survival probability of gastric cancer patients [3]. Since its initial description, ferroptosis has been implicated across pathologies ranging from neurodegeneration and ischemia–reperfusion injury to tumour suppression [4, 5]. In the cardiovascular system, mounting evidence links ferroptosis of vascular smooth muscle cells (VSMCs) to adverse vascular remodelling, fibrous‐cap thinning, plaque destabilization, aortic aneurysm progression and vascular calcification [6, 7]. VSMCs are central architects of vessel wall structure and plasticity [8]; their death or phenotypic switching (contractile → synthetic/inflammatory/osteogenic) can decisively shape lesion biology [9, 10]. Yet, the upstream stress signals that wire into the ferroptotic machinery in VSMCs remain only partially defined [11, 12].

The endoplasmic reticulum (ER) acts as a hub for proteostasis, lipid synthesis and Ca^2+^ handling [13]. Stress that perturbs ER function activates the unfolded protein response (UPR), mediated by the three canonical sensors PERK, IRE1 and ATF6 [14, 15]. Among these, the PERK–eukaryotic initiation factor 2α (EIF2α)‐activating transcription factor 4 (ATF4) arm occupies a unique position at the intersection of proteostasis and redox metabolism [16]. PERK‐dependent phosphorylation of EIF2α attenuates global translation while permitting selective translation of ATF4, a transcription factor that reprogrammes amino acid, antioxidant and stress‐response pathways [17, 18]. A critical ATF4 target in this context is ChaC glutathione‐specific γ‐glutamylcyclotransferase 1 (CHAC1), an enzyme that degrades glutathione (GSH) into 5‐oxoproline and cysteinylglycine [19, 20]. Because GSH is the obligate cofactor for GPX4, ATF4‐CHAC1‐driven GSH depletion weakens GPX4's capacity to detoxify phospholipid hydroperoxides, thereby priming cells for ferroptosis [19, 21].

This ER stress‐to‐ferroptosis axis is conceptually compelling but incompletely charted in non‐malignant vascular cells. VSMCs operate within a redox‐intense milieu—exposed to oxidized lipids, cytokines, disturbed flow and metabolic stress—where ER stress is frequently activated in vivo [22]. PERK and ATF4 activity have been detected in human and experimental atherosclerotic lesions, and ER chaperones such as GRP78 are upregulated in diseased vessels [23, 24]. However, whether and how the PERK–EIF2α–ATF4–CHAC1 signalling module *causally* drives ferroptosis in human aortic SMCs, and whether its modulation is sufficient to exacerbate or mitigate ferroptotic death, has not been systematically resolved.

Mechanistically, ferroptosis requires the convergence of three processes: (i) [25], (ii) availability of oxidizable phospholipids (amplified by ACSL4‐dependent channelling of polyunsaturated fatty acids into membranes) [26] and (iii) labile iron that catalyses Fenton chemistry and lipid peroxide propagation, often facilitated by iron‐handling pathways (e.g., transferrin receptor 1, ferritinophagy via NCOA4) [27]. In this network, lipoxygenases such as 15‐LOX (ALOX15) can further accelerate lipid peroxidation. How ER stress inputs reorganize these nodes in VSMCs—particularly GPX4 suppression downstream of ATF4–CHAC1, ACSL4/ALOX15 induction and the buildup of labile Fe^2+^—remains a key mechanistic gap with clear translational implications [28, 29, 30].

Pharmacological probes provide incisive control points to interrogate this crosstalk. Erastin, by inhibiting the cystine/glutamate antiporter system Xc−, reduces cystine import, lowers GSH synthesis and indirectly disables GPX4, robustly inducing ferroptosis [31, 32]. Buthionine sulfoximine (BSO), an inhibitor of glutamate–cysteine ligase, depletes GSH at its synthetic entry step, functionally converging on GPX4 inactivation. Conversely, salubrinal selectively inhibits EIF2α dephosphorylation, sustaining EIF2α phosphorylation and thereby potentiating ATF4 translation and its downstream programme, including CHAC1 upregulation [33]. Together, these tools allow causal testing of whether EIF2α phosphorylation dynamics and GSH availability act as upstream ‘dials’ that tune ferroptotic susceptibility in VSMCs.

Another unresolved issue is cell‐type specificity. Human aortic SMCs obtained as cell lines (HASMCs) and primary aortic smooth muscle cells (AoSMCs) can differ in basal redox buffering capacity, ER stress tone and lipid metabolic programmes [34]. Dissecting whether the ER stress–ferroptosis axis behaves similarly across these closely related VSMC sources is important for generalizability and for anticipating inter‐individual vascular responses [35]. Moreover, clarifying the *selectivity* of death programmes is essential: ferroptosis can coexist with or be misinterpreted as apoptosis or necrosis unless orthogonal readouts are applied [36, 37]. Thus, combining Annexin V/PI or TUNEL assays (for apoptosis/late death) with ferroptosis‐specific markers (GPX4 loss, ACSL4/ALOX15 induction, C11‐BODIPY oxidation, MDA accumulation and labile Fe^2+^ elevation) provides the mechanistic resolution needed to attribute cell death to ferroptosis [38].

Here, we delineate how ER stress signalling via the EIF2α–ATF4–CHAC1 axis couples to ferroptotic execution in human aortic smooth muscle cells (HASMCs). Using erastin to trigger ferroptosis, we map the temporal activation of EIF2α phosphorylation, ATF4 induction and CHAC1 upregulation, and link these events to GPX4 suppression, heightened lipid peroxidation (C11‐BODIPY shift and MDA accumulation), increased ACSL4 and ALOX15 expression, and expansion of the labile Fe^2+^ pool (Calcein‐AM quenching). We then perturb the pathway bidirectionally: BSO amplifies GSH depletion to stress the GPX4 node, whereas salubrinal sustains EIF2α phosphorylation to potentiate ATF4–CHAC1 signalling. Across HASMCs and AoSMCs, we quantify protein and transcript changes, visualize subcellular protein distributions by immunofluorescence, and benchmark death phenotypes versus apoptotic readouts to ensure mechanistic specificity.

Our study addresses three intertwined questions. **First**, does activation of the PERK–EIF2α–ATF4–CHAC1 arm constitute a *sufficient and necessary* upstream driver of ferroptosis in human aortic SMCs exposed to cystine deprivation stress? **Second**, can pharmacologic manipulation of EIF2α phosphorylation (salubrinal) or GSH biosynthesis (BSO) predictably reprogramme ferroptotic susceptibility by converging on GPX4 function and lipid peroxide handling? **Third**, are these relationships conserved across HASMC and AoSMC sources, or do basal ER stress and redox phenotypes confer differential vulnerabilities?

By integrating pathway‐level perturbations with orthogonal biochemical and imaging readouts, our work provides mechanistic evidence that ER stress signalling through the EIF2α–ATF4–CHAC1 axis is a proximal governor of ferroptotic fate in human aortic SMCs. These insights not only refine the conceptual map linking proteostasis stress to ferroptosis in vascular cells but also nominate actionable nodes—EIF2α phosphorylation dynamics, CHAC1 activity and GSH/GPX4 homeostasis—as potential therapeutic levers to mitigate ferroptosis‐related vascular injury, with implications for plaque stability, aneurysm progression and restenosis biology.

### 1.1 Study Aims

We hypothesized that (i) erastin activates the EIF2α–ATF4–CHAC1 pathway in VSMCs, leading to GSH depletion and GPX4 suppression; (ii) BSO further sensitizes cells to ferroptosis by constraining GSH synthesis, whereas salubrinal enhances EIF2α–ATF4–CHAC1 signalling and amplifies ferroptotic phenotypes; and (iii) these effects manifest consistently in HASMCs and AoSMCs and can be distinguished from apoptosis by complementary assays. We tested these hypotheses through a combined programme of Western blotting, qPCR, immunofluorescence, ferroptosis‐specific lipid oxidation probes, iron‐handling assays and quantitative image/gel densitometry.

## 2 Materials and Methods

### 2.1 Cell Culture

HASMCs (HASMCs were obtained from the Cell Bank of the Chinese Academy of Sciences Shanghai, China.) and primary HASMCs (AoSMCs were obtained from the Cell Bank of the Chinese Academy of Sciences Shanghai, China.) were cultured in Dulbecco's Modified Eagle's Medium (DMEM; Bio‐Channel，Cat. No. BC2025042, China) supplemented with 10% foetal bovine serum (FBS; Bio‐Channel, Cat. No. BC20250117, China), 100 U/mL penicillin, and 100 μg/mL streptomycin (Bio‐Channel, Cat. No. BC‐CE‐007, China) at 37°C in a humidified atmosphere containing 5% CO₂. Cells were used between passages 3 and 8. Culture medium was changed every 2–3 days, and cells were sub‐cultured upon reaching 80%–90% confluence.

### 2.2 Reagents and Treatments

Erastin (Targetmol, Cat. No. T1765, China); BSO (Targetmol, Cat. No. T5371, China); Salubrinal (Targetmol, Cat. No. 405060‐95‐9, China); Ferrostatin‐1 (Fer‐1) (Targetmol, Cat. No. T6500, China); TRIzol reagent (Invitrogen, Cat. No. 15596026, USA); CCK‐8 kit (Abbkine, Cat. No. BMU106‐CN, China); BCA Protein Assay Kit (Thermo Fisher Scientifi, Cat. No. 23225, USA); C11‐BODIPY 581/591 (Beyotime, Cat. No. S0043S, China); FerroOrange (Maokangbio, Cat. No. MX4559, China); MDA Assay Kit (Abbkine, Cat. No. KTB1050, China); TUNEL Apoptosis Detection Kit (Abbkine, Cat. No. KTA2010, China).

### 2.3 Drug Concentrations and Durations

**Erastin**: 0.5–40 μM for viability assays; 5 μM for mechanistic studies, 24 h. **BSO**: 100 μM, 24 h. **Salubrinal**: 10 μM, 24 h. **Fer‐1**: 3 μM, pre‐incubated 1 h before erastin exposure. Final DMSO concentration did not exceed 0.1% (v/v) in any treatment.

### 2.4 Primary Antibodies

(All antibodies from Immunoway unless otherwise specified).

p‐PERK (Thr980) (Cat. No. YM1055, 1:1000); PERK (Cat. No. YM8183, 1:1000); p‐EIF2α (Ser51) (Cat. No. YM8169, 1:1000); EIF2α (Cat. No. YM8313, 1:1000); GRP78/BiP (Cat. No. YM8203, 1:1000); ATF4 (Cat. No. YM8169, 1:1000); CHAC1 (Bioss, Cat. No. bs‐6795R, 1:1000); GPX4 (Cat. No. YM8430, 1:1000); ACSL4 (Cat. No. YM8287, 1:1000); ALOX15 (Cat. No. YM7564, 1:1000); β‐actin (Cat. No. YM0099, 1:5000).

### 2.5 Secondary Antibodies

HRP‐conjugated anti‐rabbit IgG (Immunoway, Cat. No. RS3208, 1:5000); HRP‐conjugated anti‐mouse IgG (Immunoway, Cat. No. RS0007, 1:5000); Alexa Fluor 488‐conjugated goat anti‐rabbit IgG (Invitroge, Cat. No. A‐11008, 1:500); Alexa Fluor 594‐conjugated goat anti‐mouse IgG (Invitroge, Cat. No. A‐11005, 1:500).

### 2.6 Cell Viability Assay

Cell viability was assessed using the CCK‐8 kit. Cells were seeded in 96‐well plates (5 × 10^3^ cells/well) and treated as described. After 24 h, 10 μL CCK‐8 solution was added to each well and incubated for 2 h at 37°C. Absorbance at 450 nm was measured using a microplate reader (Bio‐Rad, USA), and results were normalized to the control group.

### 2.7 Quantitative Real‐Time PCR (qPCR)

Total RNA was extracted with TRIzol reagent. cDNA was synthesized using a PrimeScript RT Reagent Kit (Takar, Cat. No. RR037A, Japan). qPCR was performed using SYBR Premix Ex Taq II (Takar, Cat. No. RR820A) on an ABI StepOnePlus system. GAPDH was used as an internal control, and relative gene expression was calculated by the 2^−ΔΔ*Ct*^ method. Primer sequences are listed in Table S1.

### 2.8 Western Blotting

Protein extraction was performed with RIPA buffer (Beyotime, Cat. No. P0013B) containing protease and phosphatase inhibitors. Protein concentration was measured by the BCA method. Equal amounts of protein (20–30 μg) were separated by SDS–PAGE and transferred onto PVDF membranes (Millipore, Cat. No. IPVH00010). Membranes were blocked in 5% non‐fat milk for 1 h, incubated with primary antibodies overnight at 4°C, and then with HRP‐conjugated secondary antibodies for 1 h at room temperature. Signals were developed with ECL substrate (Millipore, Cat. No. WBKLS0500) and imaged with a chemiluminescence detection system. Band intensities were quantified using ImageJ.

### 2.9 Immunofluorescence Staining

Cells grown on coverslips were fixed in 4% paraformaldehyde for 15 min, permeabilized with 0.1% Triton X‐100 for 10 min, and blocked with 5% BSA for 1 h. Primary antibodies (CHAC1, p‐EIF2α, GPX4) were applied overnight at 4°C, followed by incubation with Alexa Fluor‐conjugated secondary antibodies for 1 h. Nuclei were counterstained with DAPI (Beyotime, Cat. No. C1002). Images were captured with an Olympus IX73 fluorescence microscope.

### 2.10 Lipid Peroxidation Measurement

Lipid ROS were detected with C11‐BODIPY 581/591. Cells were incubated with 2 μM probe for 30 min, washed and imaged. The red‐to‐green fluorescence ratio was quantified. Malondialdehyde (MDA) was measured using the Beyotime kit according to the manufacturer's protocol.

### 2.11 Labile Iron (Fe^2+^) Measurement

Cells were incubated with 0.5 μM calcein‐AM for 30 min. Fluorescence quenching by Fe^2+^ was recorded at excitation/emission 488/517 nm before and after adding deferoxamine (DFO, Sigma, Cat. No. D9533). The decrease in fluorescence intensity was used to calculate Fe^2+^ levels.

### 2.12 TUNEL Assay

Apoptotic cells were identified using the Roche TUNEL kit following the manufacturer's protocol. DAPI was used for nuclear counterstaining. The proportion of TUNEL‐positive nuclei was calculated from at least five random fields per sample.

### 2.13 Statistical Analysis

All experiments were performed using three independent biological replicates (*n* = 3) unless otherwise specified. Each biological replicate contained three technical replicates. Data are presented as mean ± standard deviation (SD).

For comparisons between two groups, an unpaired two‐tailed Student's *t*‐test was applied. For multiple‐group comparisons, one‐way ANOVA followed by Tukey's post hoc test was used. *p < 0.05 was considered statistically significant. Normality and homogeneity of variance were evaluated before hypothesis testing. All statistical analyses were conducted using GraphPad Prism 9.0.

## 3 Results

### 3.1 Erastin Reduces Viability and Activates the EIF2α–ATF4–CHAC1 Pathway in Human Aortic SMCs

**FIGURE 1:**
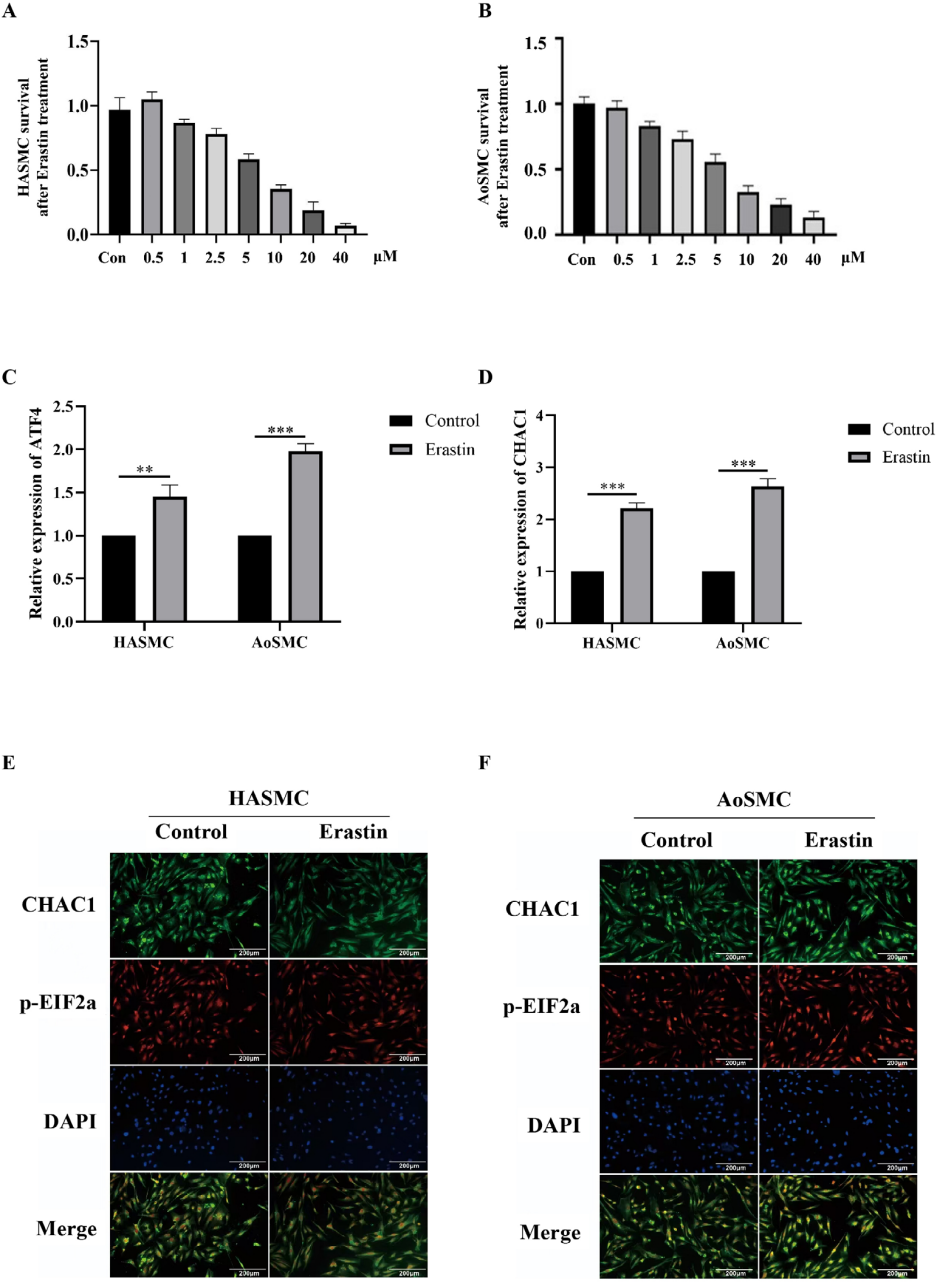
Effects of erastin on HASMCs and AoSMCs. (A) Cell viability of HASMCs after 24 h treatment with erastin at the indicated concentrations. (B) Cell viability of AoSMCs after 24 h treatment with erastin at the indicated concentrations. (C) ATF4 mRNA expression after 24 h erastin treatment. (D) CHAC1 mRNA expression after 24 h erastin treatment. (E) Immunofluorescence intensity of CHAC1 and phosphorylated EIF2α (p‐EIF2α) in HASMCs after 24 h erastin treatment. (F) Immunofluorescence intensity of CHAC1 and p‐EIF2α in AoSMCs after 24 h erastin treatment. *p < 0.05; **p < 0.01 vs. Control. Data are mean ± SD (*n* = 3).

Erastin exposure (0.5–40 μM, 24 h) led to a dose‐dependent decline in cell viability in both HASMCs and AoSMCs (Figure 1A,B). Significant reductions were evident at ≥ 5 μM (one‐way ANOVA with Tukey post hoc, *p < 0.05), with more pronounced loss at 10–40 μM (**p < 0.01 vs. Control). At 10 μM—used for downstream mechanistic assays—qPCR showed ATF4 and CHAC1 mRNA induction in HASMCs (Figure 1C,D; *p < 0.05 and **p < 0.01, respectively). Immunofluorescence revealed co‐induction and co‐localization of CHAC1 with p‐EIF2α in both cell types (Figure 1E,F). Quantification of fluorescence intensity showed an increase on the order of ~1.5–2.0‐fold relative to Control (Student's *t*‐test, *p < 0.05), consistent with activation of the EIF2α–ATF4–CHAC1 arm upon ferroptosis induction.

### 3.2 Erastin Reprogrammes Ferroptosis‐Related Proteins: EIF2α–ATF4–CHAC1 Up, GPX4 Down, ACSL4/LOX15 Up

**FIGURE 2:**
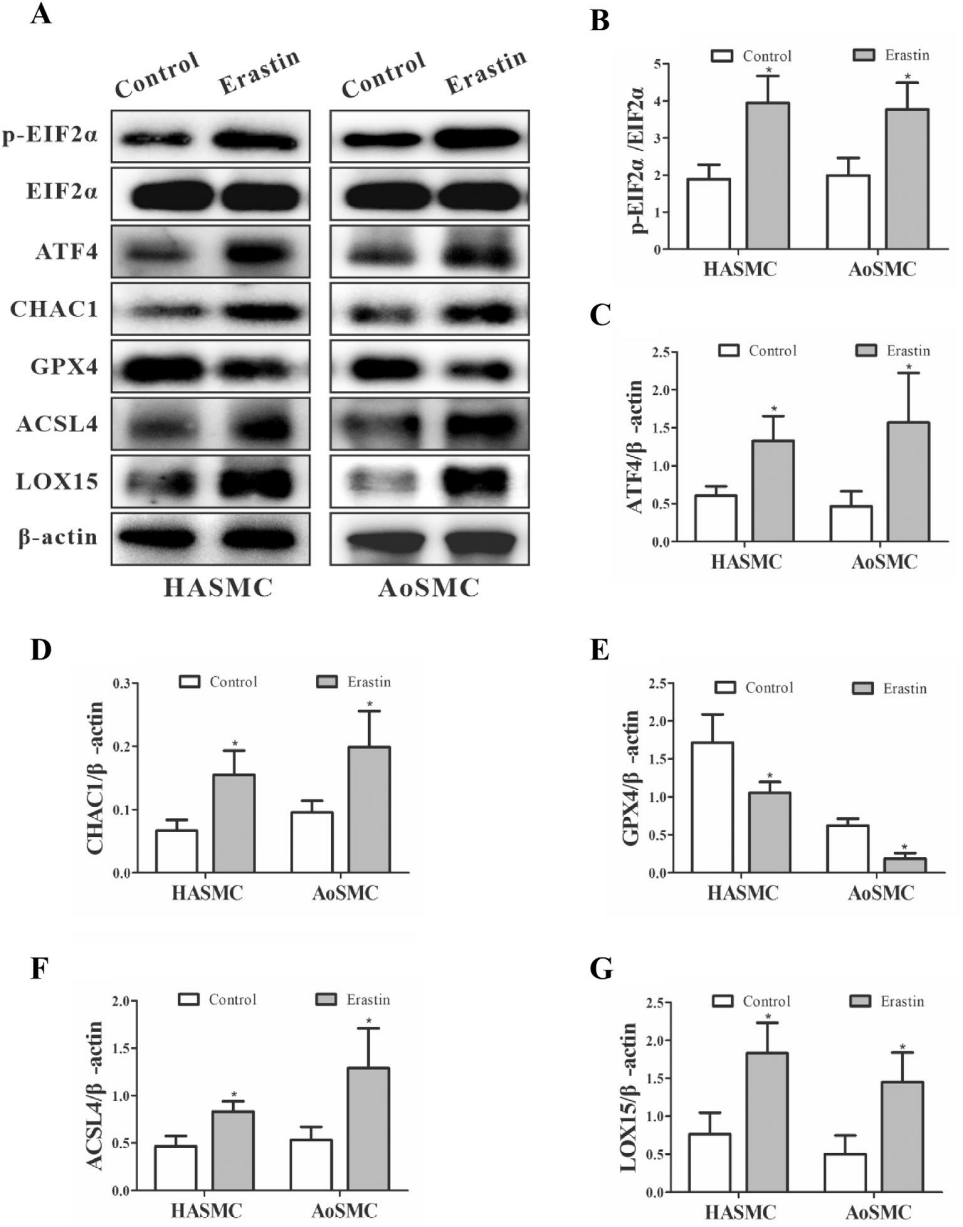
Erastin‐induced changes in ferroptosis‐related protein expression in HASMC and AoSMC cell lines. (A) Changes in Ferroptosis‐Related Protein Expression in HASMCs and AoSMCs Following Erastin Treatment. (B) Representative Western blot images showing the phosphorylation levels of eukaryotic initiation factor 2α (p‐EIF2α) in HASMCs and AoSMCs following 24 h treatment with the ferroptosis inducer erastin (10 μM), indicating activation of the EIF2α signalling axis. (C) Protein expression of activating transcription factor 4 (ATF4) under the same conditions, demonstrating ER stress‐associated transcriptional activation. (D) Expression of ChaC glutathione‐specific γ‐glutamylcyclotransferase 1 (CHAC1), a downstream effector of the EIF2α–ATF4 pathway responsible for glutathione degradation. (E) Levels of glutathione peroxidase 4 (GPX4), a key ferroptosis‐suppressing enzyme, showing a marked reduction upon erastin exposure. (F) Expression of acyl‐CoA synthetase long‐chain family member 4 (ACSL4), an essential lipid metabolism enzyme that promotes polyunsaturated fatty acid incorporation into membranes during ferroptosis. (G) Protein levels of lipoxygenase 15 (LOX15), a lipid‐peroxidizing enzyme, revealing enhanced lipid peroxidation activity after ferroptosis induction. (G) Quantitative densitometric analysis of protein band intensities for panels A–F, normalized to β‐Actin, and expressed as fold change relative to the control group. Data are presented as mean ± SD (*n* = 3 independent experiments). Statistical significance was determined by Student's *t*‐test, with *p < 0.05 and **p < 0.01 vs. Control.

Western blotting demonstrated that erastin (10 μM, 24 h) increased p‐EIF2α, ATF4 and CHAC1 protein in both HASMCs and AoSMCs (Figure 2A–C), while decreasing GPX4 (Figure 2D). Lipid‐metabolic drivers of ferroptosis, ACSL4 and LOX15 (ALOX15), were also elevated (Figure 2E,F). Densitometry (Figure 2G) indicated changes of roughly ~2.0–2.6‐fold for pathway activators (e.g., CHAC1) and ~1.5–2.0‐fold for ACSL4/LOX15, alongside a ~0.5–0.6‐fold reduction of GPX4 (all vs. Control; *p < 0.05 or **p < 0.01). Together, these data position EIF2α–ATF4–CHAC1 as a proximal ER‐stress module that converges on GPX4 loss and lipid‐peroxidation machinery during ferroptosis (Figure 2A–G).

### 3.3 Erastin Triggers GPX4 Loss and Robust Lipid Peroxidation in HASMCs and AoSMCs

**FIGURE 3:**
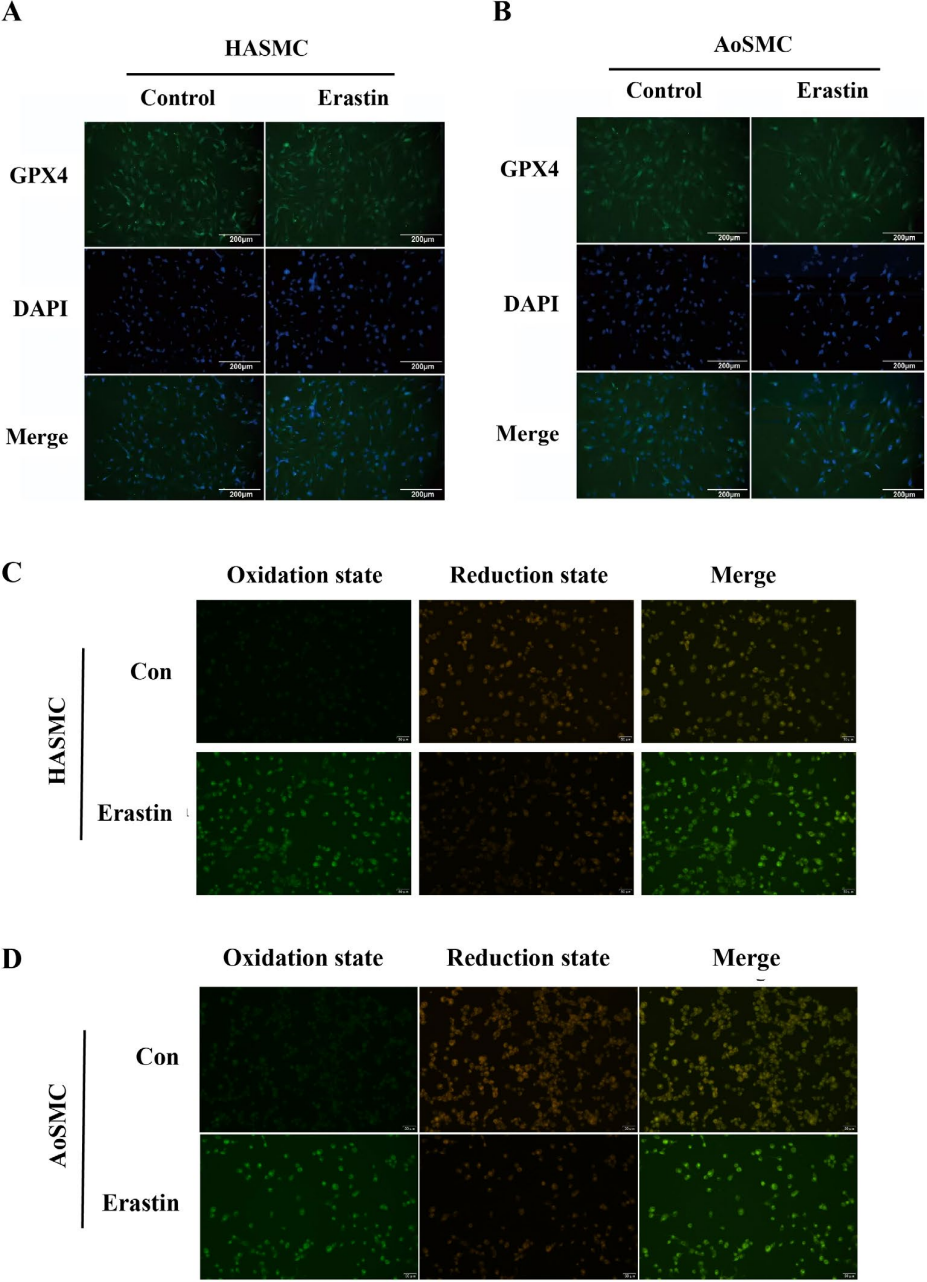
Immunofluorescence detection of GPX4 expression and lipid peroxidation in HASMCs and AoSMCs during erastin‐induced ferroptosis. (A) Representative immunofluorescence images showing glutathione peroxidase 4 (GPX4) expression in HASMCs after 24 h treatment with erastin (10 μM). Nuclei were counterstained with DAPI (blue), and GPX4 signals are shown in green, indicating a marked reduction in GPX4 abundance following ferroptosis induction. (B) GPX4 immunofluorescence in AoSMCs under the same treatment conditions, similarly demonstrating decreased GPX4 levels. (C) Lipid peroxidation in HASMCs assessed using the C11‐BODIPY 581/591 fluorescent probe, with a shift from red to green fluorescence representing oxidative modification of membrane lipids. (D) Lipid peroxidation in AoSMCs detected by C11‐BODIPY staining, confirming enhanced lipid oxidative damage upon erastin exposure.

Immunofluorescence confirmed a marked reduction of GPX4 signal intensity in both HASMCs and AoSMCs after erastin (Figure 3A,B; *p < 0.05). C11‐BODIPY 581/591 imaging showed the canonical red→green fluorescence shift, indicating oxidized phospholipid accumulation (Figure 3C,D). Quantification of the green/red ratio (oxidized/reduced states) demonstrated a significant increase vs. Control (Figure 3C,D; **p < 0.01), corroborating biochemical and WB evidence that lipid ROS is the dominant effector arm of death in this context.

### 3.4 Erastin Elevates MDA and Labile Fe^2+^; Ferrostatin‐1 Blunts Oxidative/Iron Stress; Apoptosis Remains Limited

**FIGURE 4:**
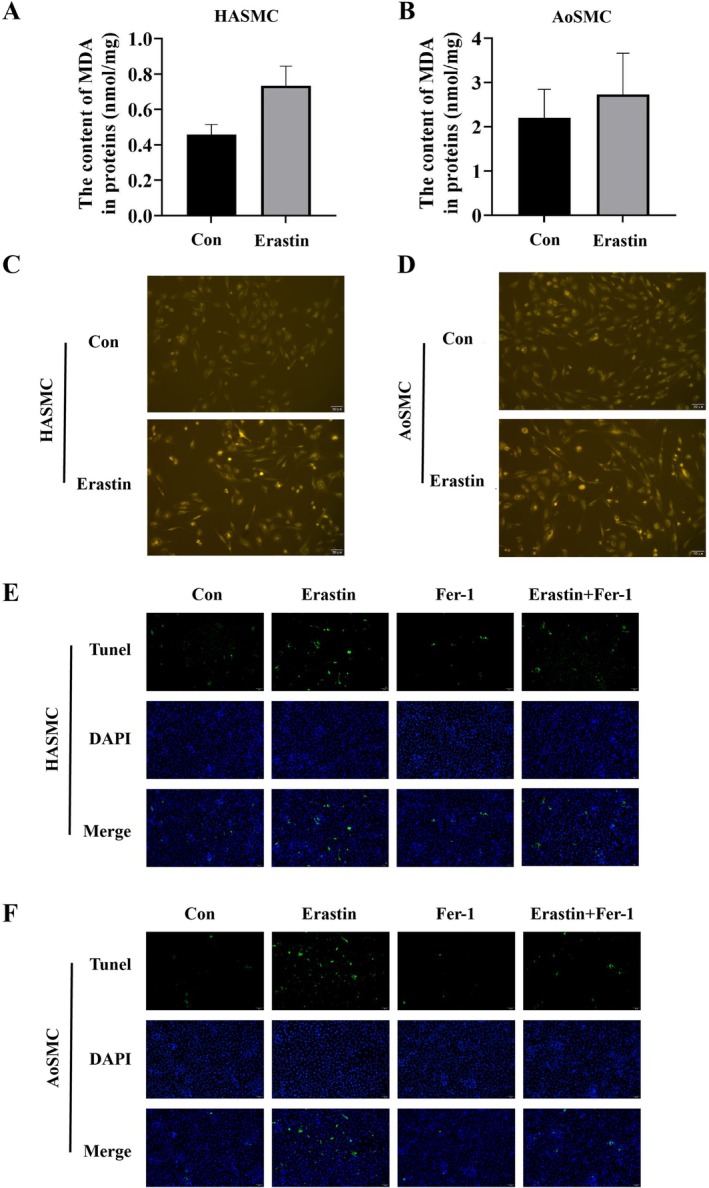
Lipid peroxidation, labile iron levels and apoptosis in HASMCs and AoSMCs during erastin‐induced ferroptosis. (A) Quantification of malondialdehyde (MDA) levels, a key end product of lipid peroxidation, in HASMCs following 24 h treatment with erastin (10 μM), showing a significant increase compared with the control group. (B) MDA levels in AoSMCs under the same treatment conditions, demonstrating a similar elevation in lipid peroxidation. (C) Measurement of intracellular labile ferrous iron (Fe^2+^) in HASMCs using the Calcein‐AM fluorescence quenching assay, indicating iron accumulation upon erastin exposure. (D) Fe^2+^ levels in AoSMCs measured as in panel C, confirming increased intracellular iron availability during ferroptosis. (E) Detection of apoptotic cells in HASMCs by TUNEL (terminal deoxynucleotidyl transferase dUTP nick end labelling) staining, showing limited apoptosis in the context of ferroptotic cell death. (F) TUNEL assay in AoSMCs, revealing a similar pattern of apoptotic cell distribution. Data are presented as mean ± SD (*n* = 3 independent experiments). *p < 0.05, **p < 0.01 vs. Control.

Biochemical readouts aligned with imaging: MDA (lipid peroxidation end‐product) was elevated by erastin in both HASMCs and AoSMCs (Figure 4A,B; HASMCs ~0.85 vs. 0.27 nmol/mg protein in Control, Figure 4A; *p < 0.01). The labile Fe^2+^ pool (calcein‐AM quenching) increased significantly under erastin (Figure 4C,D; **p < 0.01), consistent with iron‐dependent lipid peroxidation. Notably, ferrostatin‐1 (Fer‐1, 1 μM) partially restored both readouts toward baseline (Erastin + Fer‐1 vs. Erastin, Figure 4C,D, right panels; *p < 0.05 to **p < 0.01), supporting ferroptosis as the predominant death programme. TUNEL assays detected only modest apoptosis relative to ferroptotic markers (Figure 4E,F), indicating that apoptosis is not the principal mechanism under these conditions (ns or *p* < 0.05 vs. Control depending on cell type; Figure 4E,F).

### 3.5 Pharmacologic GSH Depletion by BSO Potentiates ER‐Stress Signalling and GPX4 Loss

**FIGURE 5:**
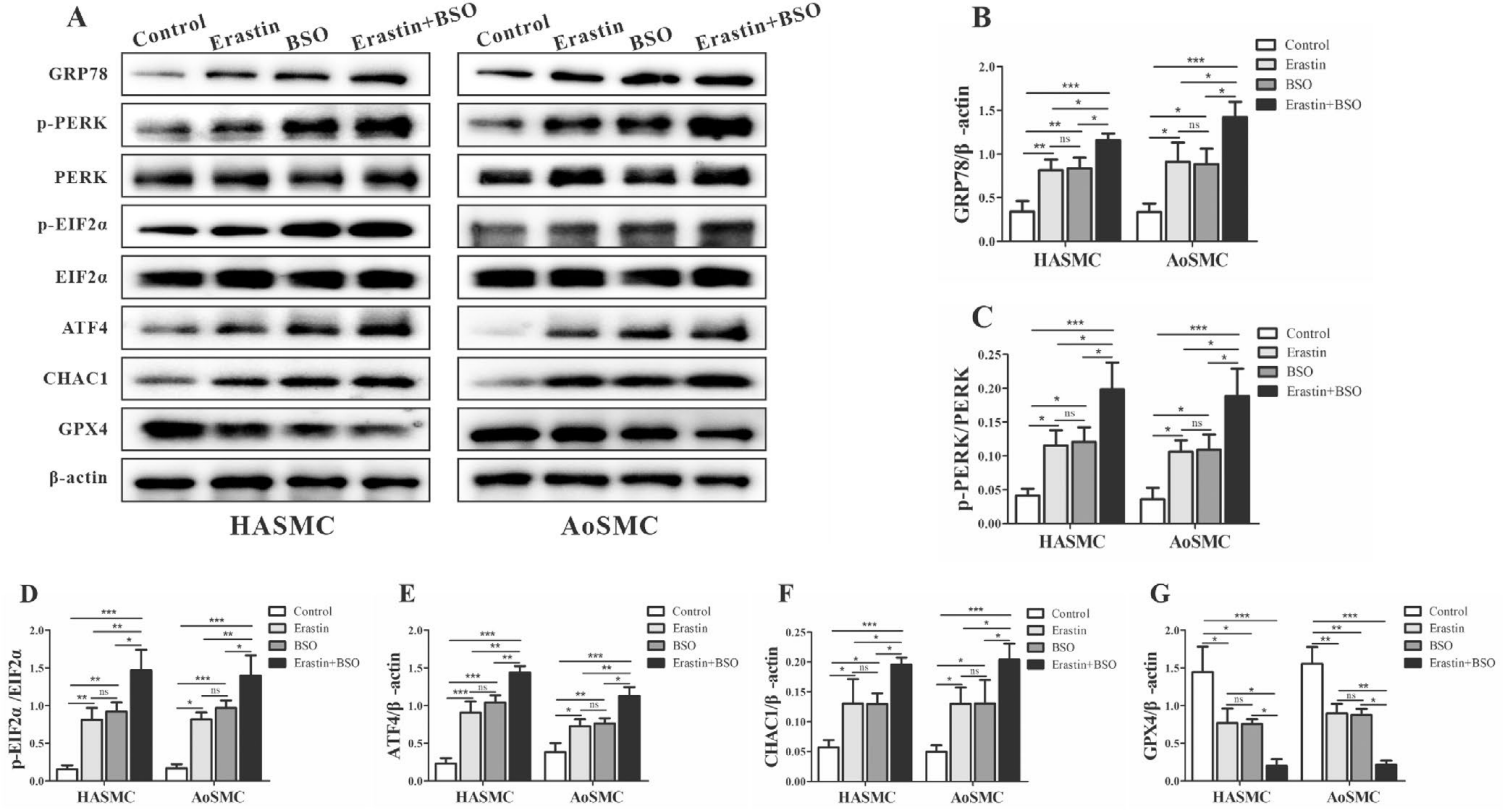
Effects of glutamate–cysteine ligase inhibition by BSO on ferroptosis‐related protein expression in HASMCs and AoSMCs. (A) Alterations in Ferroptosis‐Related Protein Expression Following Glutamate–Cysteine Ligase Inhibition by BSO in HASMCs and AoSMCs. (B) Glucose‐regulated protein 78 (GRP78) expression in both cell types, indicating upregulation of this classical ER chaperone in response to glutathione depletion. (C) Western blot analysis of phosphorylated protein kinase RNA‐like ER kinase (p‐PERK) in HASMCs and AoSMCs after 24 h treatment with buthionine sulfoximine (BSO, 100 μM), showing marked activation of the PERK branch of the ER stress response. (D) Phosphorylated eukaryotic initiation factor 2α (p‐EIF2α) protein levels under the same conditions, demonstrating increased phosphorylation downstream of PERK activation. (E) Activating transcription factor 4 (ATF4) protein expression, significantly elevated following BSO treatment, consistent with EIF2α pathway activation. (F) ChaC glutathione‐specific γ‐glutamylcyclotransferase 1 (CHAC1) protein levels, showing pronounced induction, suggesting enhanced degradation of intracellular glutathione. (G) Glutathione peroxidase 4 (GPX4) expression, markedly decreased in both HASMCs and AoSMCs, reflecting impaired antioxidant defence and promotion of ferroptosis. Densitometric quantification of protein bands from panels (A–F), normalized to β‐Actin and expressed as fold change relative to control, confirming statistically significant changes in response to BSO. Data are presented as mean ± SD (*n* = 3 independent experiments). *p < 0.05, **p < 0.01 vs. Control.

We next asked whether further GSH limitation amplifies ferroptosis. BSO (100 μM, 24 h) alone elevated p‐PERK, p‐EIF2α, GRP78, ATF4, and CHAC1, and reduced GPX4 (Figure 5A–F). Co‐treatment (Erastin + BSO) produced additive to synergistic changes versus erastin alone—for example, p‐EIF2α/ATF4/CHAC1 rising by an additional ~1.3–1.6× over erastin and GPX4 dropping to ~0.4–0.6× Control (densitometry, Figure 5G; *p < 0.05 or **p < 0.01 for pairwise comparisons). These data indicate that constraining GSH biosynthesis via GCL inhibition drives the EIF2α–ATF4–CHAC1 → GPX4 axis further into a pro‐ferroptotic state in both HASMCs and AoSMCs (Figure 5A–G).

### 3.6 Sustaining EIF2α Phosphorylation With Salubrinal Amplifies ATF4–CHAC1 Signalling and Depresses GPX4

**FIGURE 6:**
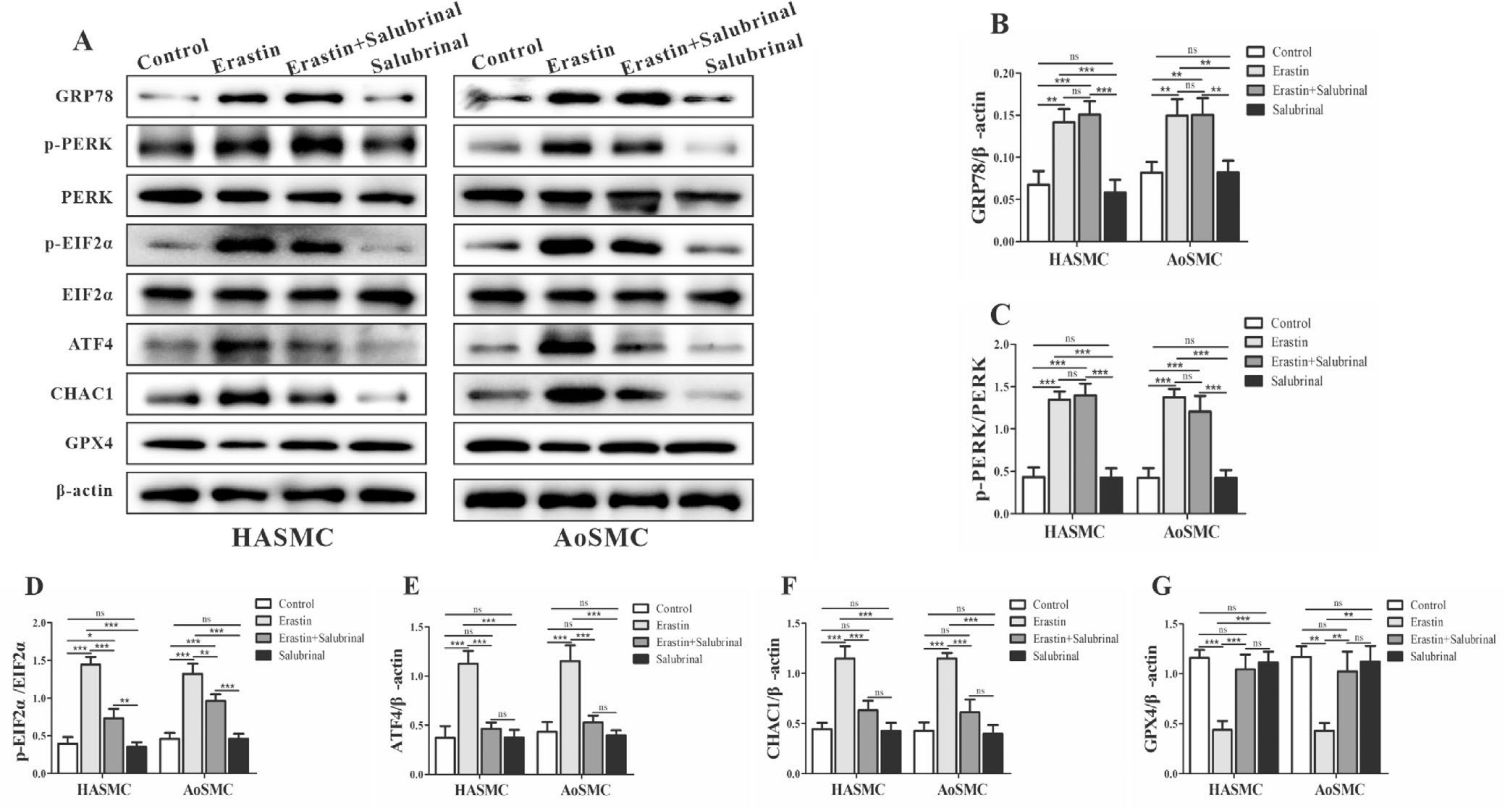
Effects of the selective eIF2α dephosphorylation inhibitor salubrinal on ferroptosis‐related protein expression in HASMCs and AoSMCs. (A) Alterations in Ferroptosis‐Related Protein Expression Following Treatment with the eIF2α Dephosphorylation Inhibitor Salubrinal in HASMCs and AoSMCs. (B) Glucose‐regulated protein 78 (GRP78) expression, reflecting the activation status of ER chaperone machinery under conditions of altered eIF2α signalling. (C) Western blot analysis of phosphorylated protein kinase RNA‐like ER kinase (p‐PERK) in HASMCs and AoSMCs after 24 h treatment with salubrinal (25 μM), showing modulation of the ER stress response via inhibition of eIF2α dephosphorylation. (D) Phosphorylated eukaryotic initiation factor 2α (p‐EIF2α) protein levels, demonstrating sustained phosphorylation in both cell types following salubrinal treatment. (E) Activating transcription factor 4 (ATF4) protein levels, upregulated in response to persistent eIF2α phosphorylation, consistent with activation of downstream stress signalling. (F) ChaC glutathione‐specific γ‐glutamylcyclotransferase 1 (CHAC1) protein expression, indicating potential enhancement of glutathione degradation following eIF2α pathway activation. (G) Glutathione peroxidase 4 (GPX4) protein levels, showing a trend toward reduction, suggestive of compromised antioxidant defence and potential promotion of ferroptotic processes. Densitometric quantification of protein bands from panels (A–F), normalized to β‐Actin and expressed as fold change relative to control, confirming significant alterations in ferroptosis‐related proteins in response to salubrinal treatment. Data are presented as mean ± SD (*n* = 3 independent experiments). *p < 0.05, **p < 0.01 vs. Control.

Because EIF2α phosphorylation gates ATF4 translation, we inhibited EIF2α dephosphorylation with salubrinal (25 μM, 24 h). Salubrinal maintained elevated p‐EIF2α and increased ATF4 and CHAC1, with a concomitant reduction of GPX4 (Figure 6A–F). When combined with erastin, salubrinal further enhanced pathway activation versus erastin alone—densitometry indicated ~1.3–1.8× increases in p‐EIF2α/ATF4/CHAC1 and ~0.5–0.7× GPX4 relative to Control (Figure 6G; *p < 0.05 to **p < 0.01). These results support a causal position for EIF2α phosphorylation dynamics in modulating ferroptotic susceptibility in human aortic SMCs (Figure 6A–G).

### 3.7 Erastin Induces Parallel Upregulation of Ferroptosis‐ and ER Stress‐Related Molecular Markers in VSMCs

**FIGURE 7:**
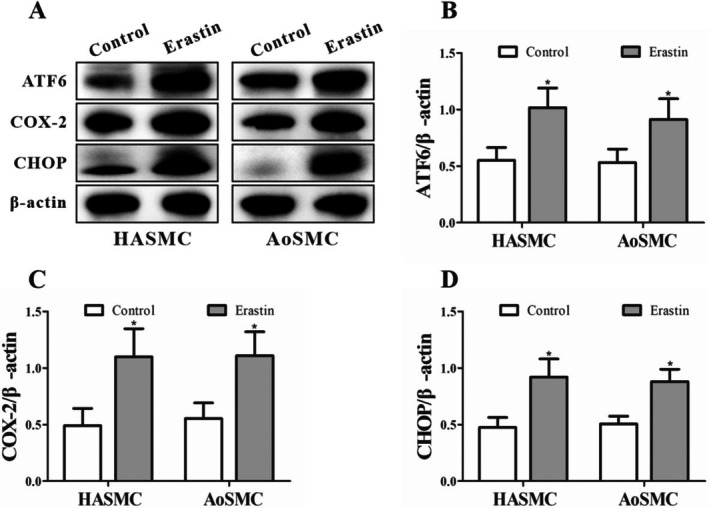
Erastin‐induced changes in ferroptosis‐ and endoplasmic reticulum stress‐related protein markers in vascular smooth muscle cells. (A) Representative Western blot images showing the protein expression levels of ATF6, CHOP and COX‐2 in HASMCs and AoSMCs after treatment with erastin or vehicle control. β‐Actin was used as a loading control. (B–D) Quantitative densitometric analysis demonstrated that erastin treatment resulted in a marked upregulation of ATF6 (B), COX‐2 (C) and CHOP (D) protein expression in both HASMCs and AoSMCs compared with their respective control groups. Data are expressed as mean ± SEM. *p < 0.05 vs. Control.

Densitometric quantification revealed that erastin treatment significantly increased the expression of COX‐2, a well‐established molecular marker associated with the occurrence of ferroptosis, indicating activation of ferroptotic processes in both HASMCs and AoSMCs. In parallel, the expression levels of ATF6 and CHOP, two canonical markers of ER stress, were also markedly elevated compared with control groups, reflecting the induction of ER stress (Figure 7). Importantly, the upregulation of COX‐2 and that of ATF6 and CHOP were analysed as independent molecular events representing distinct cellular stress responses. Similar directional trends were observed in both cell types, although the magnitude of change varied between HASMCs and AoSMCs.

## 4 Overall Synthesis

Across HASMCs and AoSMCs, erastin decreases viability, activates EIF2α–ATF4–CHAC1, suppresses GPX4, and elevates lipid peroxidation and Fe^2+^, with Fer‐1 rescue confirming ferroptosis. BSO shifts cells toward ferroptosis by tightening GSH supply, while salubrinal does so by sustaining EIF2α phosphorylation—both amplify ATF4–CHAC1 signalling and depress GPX4 (Figures 1, 2, 3, 4, 5, 6; all key comparisons *p < 0.05, most **p < 0.01). These pathway‐level perturbations place EIF2α phosphorylation/CHAC1‐mediated GSH erosion and GPX4 insufficiency at the centre of the ferroptotic decision in human aortic SMCs.

## 5 Discussion

Our data identify ER‐stress signalling through the EIF2α–ATF4–CHAC1 axis as a proximal driver of ferroptosis in HASMCs. Exposure to erastin decreased viability in both HASMCs and AoSMCs and concomitantly activated EIF2α phosphorylation with induction of ATF4 and CHAC1 at the transcript and protein levels, as visualized by immunofluorescence colocalization (Figure 1A–F). This stress‐response configuration coincided with a characteristic ferroptotic effector signature—suppression of GPX4 together with elevations in ACSL4 and ALOX15—demonstrated by Western blotting and quantitative densitometry (Figure 2A–G). Imaging and biochemical readouts independently converged on this conclusion: GPX4 signal intensity was reduced in both cell types by immunofluorescence, the C11‐BODIPY probe revealed a robust red‐to‐green shift indicative of lipid peroxide accumulation, and MDA content increased alongside expansion of the labile Fe^2+^ pool (Figures 3A–D and 4A–D). Although TUNEL positivity modestly increased, ferrostatin‐1 attenuated lipid peroxidation and iron readouts, supporting ferroptosis—not apoptosis—as the dominant death programme under these conditions (Figure 4C–F).

Taken together, these findings support a mechanistic model wherein PERK‐dependent phosphorylation of EIF2α selectively enhances translation of ATF4, which transcriptionally upregulates CHAC1; CHAC1 then degrades intracellular glutathione, weakening GPX4‐mediated detoxification of phospholipid hydroperoxides and lowering the threshold for ferroptosis. In parallel, ACSL4 channels polyunsaturated fatty acids into membrane phospholipids, while ALOX15 and labile Fe^2+^ catalyse and propagate lipid peroxidation, driving cells past a commitment point to ferroptotic death (Figures 2D–F, 3C,D, and 4A–D). The pharmacological triangulation further strengthens causality: restricting GSH biosynthesis with BSO amplified PERK→EIF2α → ATF4 → CHAC1 signalling and further depressed GPX4, with additive effects when combined with erastin (Figure 5A–G); sustaining EIF2α phosphorylation with salubrinal similarly elevated ATF4 and CHAC1 and reduced GPX4, especially in combination with erastin (Figure 6A–G). Across assays, most pairwise comparisons versus control or single‐agent conditions reached statistical significance (*p < 0.05 or **p < 0.01), and the densitometric changes for pathway activators typically ranged around 1.3–2.6‐fold with GPX4 decreasing to ~0.4–0.7× of control levels, consistent with a coherent pathway‐to‐phenotype link.

These results align with the context‐dependent nature of the integrated stress response in vascular cells. While transient EIF2α phosphorylation can be cytoprotective in acute proteotoxic stress by curbing global translation [39, 40], sustained activation in a redox‐intense milieu appears to tip the balance toward ATF4–CHAC1‐mediated erosion of the GSH/GPX4 axis and, consequently, ferroptosis. The observation that salubrinal—often protective in protein‐misfolding paradigms—exacerbated ferroptotic signalling when cystine/GSH supply was constrained (Figure 6A–G) underscores this duality and cautions that ISR‐targeting strategies may have divergent effects depending on redox context and lipid peroxidation pressure. Importantly, the consistency of responses in HASMCs and primary AoSMCs suggests that the ER‐stress‐to‐ferroptosis conduit is not an artefact of a single cell source but a shared vulnerability of human aortic SMCs.

From a vascular biology standpoint, positioning EIF2α–ATF4–CHAC1 upstream of GPX4 insufficiency and lipid ROS has implications for diseases in which smooth muscle integrity governs plaque stability and aneurysm wall strength [41, 42]. Therapeutic approaches that preserve GSH/GPX4 tone (e.g., supporting cystine import or GSH synthesis, or using direct ferroptosis inhibitors), limit labile iron expansion, or restrain excessive EIF2α → ATF4 → CHAC1 signalling could, in principle, protect VSMCs in oxidizing microenvironments [21]. Conversely, selective enhancement of ferroptosis might be leveraged where elimination of maladaptive SMC clones is desirable [43], though such an approach would demand precise spatial and temporal control to avoid compromising fibrous‐cap resilience. The partial rescue by ferrostatin‐1 in our system (Figure 4C,D, right panels) offers a functional foothold for such considerations.

We acknowledge several limitations that temper generalization and point to clear next steps. First, although pharmacological modulation provides causal support, genetic perturbations would sharpen specificity: CHAC1 or ATF4 loss‐of‐function, non‐phosphorylatable (S51A) or phosphomimetic EIF2α mutants, and GPX4 overexpression or selenium supplementation could validate pathway positions and rescue points with higher resolution. Second, we quantified GRP78 but did not dissect IRE1/XBP1s or ATF6 branches; mapping their contributions could reveal compensatory circuits that intersect with lipid metabolism. Third, direct assessment of system Xc − (SLC7A11) activity and sulfur‐metabolite flux would connect erastin's proximal target to downstream GSH/GPX4 insufficiency more explicitly. Fourth, iron‐handling modules (TFR1, ferroportin, ferritin heavy/light chains and NCOA4‐dependent ferritinophagy) were not profiled; these pathways likely shape the magnitude of labile Fe^2+^ expansion observed here. Finally, lipidomics to define oxidized PE species, ultrastructural confirmation by transmission electron microscopy, and in vivo validation in atherosclerosis or aneurysm models would extend mechanistic insights toward translational relevance.

In conclusion, by integrating pathway activation, effector suppression, biochemical and imaging readouts, and bidirectional pharmacology, we demonstrate that the EIF2α–ATF4–CHAC1 axis is a decisive conduit from ER stress to ferroptotic execution in HASMCs. The convergence of GSH depletion and sustained EIF2α phosphorylation on GPX4 insufficiency, lipid peroxidation and iron‐dependent propagation—together with ferroptosis blockade by ferrostatin‐1—provides a coherent framework for understanding VSMC loss under oxidative stress. These findings nominate tractable nodes for intervention to preserve vascular integrity and offer a roadmap for mechanistic and translational studies that target ER‐stress/ferroptosis crosstalk in vascular disease.

## 6 Conclusion

This study identifies the PERK–EIF2α–ATF4–CHAC1 axis as a decisive conduit linking ER stress to ferroptotic execution in HASMCs. Erastin reduced cell viability while activating EIF2α phosphorylation and inducing ATF4 and CHAC1, coincident with GPX4 suppression, ACSL4/ALOX15 upregulation, robust lipid peroxidation (C11‐BODIPY shift; increased MDA), and expansion of the labile Fe^2+^ pool; ferrostatin‐1 mitigated these changes, confirming ferroptosis as the dominant death programme. These findings were consistent across HASMCs and AoSMCs, underscoring a shared vulnerability of human aortic SMCs to ER stress‐driven ferroptosis.

Pharmacological triangulation further clarifies pathway control points: restricting glutathione biosynthesis with BSO and sustaining EIF2α phosphorylation with salubrinal both amplified ATF4–CHAC1 signalling and deepened GPX4 insufficiency, thereby heightening ferroptotic susceptibility. Collectively, the data position EIF2α phosphorylation dynamics and CHAC1‐mediated GSH erosion—converging on GPX4 dysfunction—as proximal nodes that govern the ferroptotic threshold in vascular smooth muscle. Conceptually and translationally, these results nominate actionable strategies to preserve SMC integrity in oxidizing vascular niches: maintain cystine/GSH–GPX4 homeostasis, restrain excessive EIF2α → ATF4 → CHAC1 signalling, and limit labile iron availability. Future work coupling genetic perturbation with lipidomics and in vivo validation should refine this mechanistic framework and guide targeted interventions for ferroptosis‐related vascular injury.

## Author Contributions

**Yiqi Jin:** writing – original draft, validation. **Changwen Fang:** writing – original draft, formal analysis, software, data curation, conceptualization, visualization, project administration. **Wenbin Wang:** funding acquisition, writing – original draft, writing – review and editing, methodology, supervision, resources. **Xiaolong Du:** methodology. **Yadan Wang:** methodology.

## Funding

This work was supported by Key Project of Anhui Provincial Department of Education (Grant 2024AH050802), Key Project of Anhui Translational Medicine Research Institute (Grant 2022zhyx‐B08), Natural Science Foundation of Anhui Medical University (Grant 2023GMFY02).

## Conflicts of Interest

The authors declare no conflicts of interest.

## Supporting information


**Table S1:** Supplementary Table.

## Data Availability

The data that support the findings of this study are available from the corresponding author upon reasonable request.
