# Supplementary material for: EIF2α–ATF4–CHAC1 Signalling Links ER Stress to Ferroptosis in Human Aortic Smooth Muscle Cells: Mechanistic Insights and Therapeutic Implications
Source: J Cell Mol Med. 2026 May 2;30(9):e71104. doi: 10.1111/jcmm.71104 (PMC13135238; doi:10.1111/jcmm.71104)
Supplement: Supplementary file 1 — Table S1: Supplementary Table. [file JCMM-30-e71104-s001.docx]

Supplementary table 1

| GAPDH-F | GAGAAGGCTGGGGCTCATTT |
| --- | --- |
| GAPDH-R | GTCAAAGGTGGAGGAGTGGG |
| ATF4-F | ACAGCAAGGAGGATGCCTTC |
| ATF4-R | GACCCACAGAGAACACCTGG |
| CHAC1-F | GAGTCTGCAGCCCCGAAC |
| CHAC1-R | CTCGGTGGGGCAGAAGCAG |
